# Nek2 Is a Novel Regulator of B Cell Development and Immunological Response

**DOI:** 10.1155/2014/621082

**Published:** 2014-11-17

**Authors:** Zhimin Gu, Wen Zhou, Junwei Huang, Ye Yang, Erik Wendlandt, Hongwei Xu, Xiao He, Guido Tricot, Fenghuang Zhan

**Affiliations:** ^1^Department of Internal Medicine, University of Iowa City, Carver College of Medicine, Iowa City, IA 52242, USA; ^2^Institute of Cancer Research, School of Basic Medical Sciences, Southern Medical University, Guangzhou 510515, China; ^3^Department of Pathology, The University of Utah, Salt Lake City, UT 84102, USA

## Abstract

The serine/threonine kinase Nek2 is commonly found upregulated in a wide variety of neoplasms including diffuse large B cell lymphoma and multiple myeloma. High expression of Nek2 is implicated in the induction of chromosomal instability, promotion of cell proliferation, and drug resistance in tumor cells as well as a marker for poor clinical outcomes. Despite its well recorded involvement in chromosomal instability and neoplastic growth, little is known about the involvement of Nek2 in B cell development. Here we report the development of a transgenic mouse line with conditional expression of Nek2 in the B cell lineage and the effects it has on the development of B cells. Interestingly, we found that the overexpression of Nek2 does not induce spontaneous tumor formation within the transgenic mice up to 24 months after induction. Instead, overexpression of Nek2 in the B cell lineage affects the development of B cells by increasing the proportion of immature B cells in the bone marrow and decreasing B-1 B cells in peritoneal cavity. Furthermore, Nek2 transgenic mice develop spontaneous germinal centers and exhibit an enhanced T cell dependent immune response. Altogether, our data demonstrates a novel role for Nek2 in regulating B cell development and the immune response.

## 1. Introduction

Nek2 is a serine/threonine kinase important for the regulation of the cell cycle, gene expression, and maintenance of centrosomal structure and function [1–3]. Nek2 belongs to the Never in Mitosis A or NIMA-related family of kinases or Neks [3]. The Nek family consists of 11 members, with eleven reported in mammals [3]. Of the mammalian Neks, Nek2 shares the greatest sequence similarity to NIMA [3]. The regulation of Nek2 is strictly controlled throughout the cell cycle with highest expression during S and G_2_ phase where it regulates centrosomal function [1, 4]. Furthermore, noncentrosomal Nek2 expression peaks from S phase to G_2_ phase, cytoplasmic Nek2 is highest in G_1_, and nuclear Nek2 is highest in S phase and G_2_ phase [5, 6]. Studies have shown that Nek2 cooperates with the Hippo pathway to regulate centrosomal disjunction through the disassembly of the two linker proteins, C-Nap1 and rootletin [7]. Upon partial inhibition of the kinesin-5 Eg5 motor, Nek2 becomes essential for regulating the formation of the bipolar spindles through its interaction with Sav-1 and Mst2 [7]. Interestingly, a recent report demonstrated that Nek2 also regulates gene expression through the phosphorylation of the splicing factor SRSF1 [8].

Nek2 regulation is important for proper cell cycle progression and segregation of chromosomes during mitosis. Dysregulation of Nek2 has dire consequences for the cell and rearrangements and amplifications of chromosome 1q32 are commonly observed in various types of neoplastic growth [9–11]. A study by our group has shown that high* Nek2* expression is correlated with poor prognosis in myeloma patients due to increased activity of drug efflux pumps as the result of Nek2 activating the AKT pathway [9]. Furthermore, suppression of Nek2 results in the decreased viability of cancer cells in solid cancers, like breast cancer [12], non-small cell lung cancer [13], colorectal cancer [14], ovarian cancer [15], and liquid cancers like myeloma [9]. Our understanding of Nek2's importance in regulation of the cell cycle, disease progression, and drug resistance has improved dramatically leading to the development of Nek2 as a poor prognostic marker in myeloma and other cancers. However, the alternative functions of Nek2 remain elusive due to lack of animal models, which prompts research to develop a full understanding of the importance of Nek2* in vivo*.

Mechanistically, elevated expression of Nek2 induces chromosomal instability through the promotion of centrosome amplification and aneuploidy. Nek2 functions downstream of oncogenic Ras signaling and cooperates with cyclin D1/CDK4 resulting in centrosomal amplification [16]. Furthermore, phosphorylation and stabilization of *β*-catenin by Nek2 function downstream of PLK4 and regulate centrosomal separation [17]. Nek2 kinase activity also modulates chromosome alignment and signaling of the spindle assembly checkpoint by phosphorylating HEC1, a component of the Ndc80 complex [18]. The interaction between HEC1 and Nek2 provides an attractive therapeutic target within myeloma. Small molecules, such as INH (inhibitor for Nek2 and HEC1 binding which disrupt HEC1/Nek2 interaction), may ablate tumor progression by triggering Nek2 degradation through a death-trap mechanism [19]. The multifaceted role of Nek2 within the cell makes it an attractive target for therapeutic design and warrants further research to fully elucidate its mechanisms of action.

B cell development is a highly regulated and orchestrated process. B cell precursors are derived from hematopoietic stem cells in the bone marrow. B cells sequentially develop through four stages arising from hematopoietic stem cells and include early pro-B cells, late pro-B cells, pre-B cells, and immature B cells. Immature B cells exit from the bone marrow and migrate to the spleen and other peripheral lymph nodes, where they differentiate into peripheral mature B cells and plasma cells [20]. The change in gene expression patterns as B cells progress through development allows researchers to identify each B cell developmental stage. For example, immature B cells exhibit the unique profile (B220^+^ CD43^+^, BP-1^−^, and CD24^+^). It is the unique expression patterns of the developmental intermediates that allow for elucidation of the effects Nek2 has on B cell development and identification of the mechanism of action.

Nek2 expression is an important mediator in multiple myeloma, an end stage of B cell neoplasm, so we queried the importance of Nek2 in B cell development. Here we employed a transgenic mouse model to explore* in vivo* functions of Nek2 in the context of B cell lineage. Our data defines a new role for Nek2 in regulating B cell development and the T cell dependent immune response.

## 2. Material and Methods

### 2.1. Generation of Nek2 Transgenic Mice

Mouse Nek2 (accession: NM_010892.3) was cloned from the testis of a C57BL6 background mouse. The complete sequenced coding region was inserted into the pTraffic vector (a gift from Haojie Huang, Masonic Cancer Center, University of Minnesota, Minneapolis, MN). An 8.5 kb DNA fragment containing a CAG promoter-loxp-DsRed2-SV40 poly A-loxp-Nek2-IRES-GFP sequence was injected into C57BL6 × CBA hybrid embryos in the genetics core at the University of Utah. Founders were preselected with DsRed2 and genotyped with primers against the region of Nek2-IRES and GFP (F1, CAC GGG GAA AGC AAA GAG AAC; F2, CCT CAC ATT GCC AAA AGA CG; F3, CAC ATG AAG CAG CAC GAC TT; F4, AGT TCG CCT TGA TGC CGT TC; Cre forward GCG GTC TGG CAG TAA AAA CTA TC; Cre reverse GTG AAA CAG CAT TGC TGT CAC TT). Two lines were selected and backcrossed to a C57BL6 background for at least ten generations. Mice positive for the transgene were bred with CD19-Cre mice to selectively induce the expression of Nek2 in the B cell lineage. All mouse experiments were performed with protocols approved by the Institutional Animal Use and Care Committee of the University of Iowa.

### 2.2. Real-Time PCR

Splenocytes were sorted by flow cytometry using anti-mouse CD19 antibody (BioLegend, San Diego, CA). Cells were washed with 1x PBS and mRNA was extracted using an RNeasy RNA extraction kit (Qiagen, Valencia, CA) following the manufactures protocol. Messenger RNA was reverse-transcribed to cDNA using an iScript Supermix synthesis kit (Bio-Rad, Hercules, CA) following the manufactures recommendations. Gene expression was quantified by RT-qPCR as described previously [21]. The primers for detecting expression of Nek2 are TTC CAT CCT CAG CCA TGA AGA and CCT GCA CTT GGA CTT GGC AA.

### 2.3. B Cell Subpopulation Analysis by Flow Cytometry

Sex matched mice at the age of 6–8 weeks were sacrificed by CO_2_ asphyxiation. Peritoneal cavity cells were collected by rinsing with FACS buffer (1x PBS plus 2% fetal bovine serum). Cells from the bone marrow and spleen were lysed with a red blood cell lysis buffer to remove red blood cells and filtered through a 70 *μ*M cell strainer (BD Biosciences, Franklin Lakes, NJ). After washing with cold 1x PBS, cells were counted by trypan blue staining. One million cells were resuspended in 100 *μ*L FACS buffer and labeled with 1 *μ*L antibodies of B220-APC, CD43-FITC, BP-1-PE, CD24-PerCP/Cy5.5, CD19-APC-Cy7, IgM-PE, IgD-PerCP/Cy5.5, IgM-PerCP/Cy5.5, CD93-APC, CD23-PE, CD21-FITC, CD5-PerCP (BioLegend, San Diego, CA). Cell subpopulations were analyzed on BD LSR II violet at The University of Iowa flow cytometry facility.

### 2.4. Mouse Immunization

For T cell dependent immunization, mice were immunized with alum participated TNP-KLH (Biosearch Technologies. Inc., Petaluma, CA) at day 0 and boost at day 14. Mice were bled at day 0, 7, 14, 21, 28. For T cell independent immunization, mice were immunized with TNP-Ficoll (Biosearch Technologies, Novato, CA) at day 0 and bled at day 10.

### 2.5. ELISA

ELISA plates were coated with TNP-BSA overnight in a sodium carbonate buffer and then blocked with 0.5% BSA in PBS for one hour at room temperature. Sera were diluted with blocking buffer and incubated in 96 well plates at 4°C overnight. In each plate, TNP-specific standard IgG1, IgM (BD Biosciences, Franklin Lakes, NJ), and IgG3 (a gift from Dr. Thomas Waldschmidt, University of Iowa, Iowa City, IA) were added accordingly. Plates were washed and incubated with an appropriate HRP-conjugated isotype specific anti-mouse Ig antibody (BD, Franklin Lakes, NJ). Plates were washed, developed, and stopped. Absorbance of 450 nm was detected by Microplate reader (BioTek, Winooski, VT).

### 2.6. Western Blotting

FACS sorted cells were pelleted and directly lysed with 2% SDS lysis buffer, boiled for 10 minutes, and quantified. Western blots were performed as previously described, with antibodies of Nek2 (Santa Cruz Biotechnology, Santa Cruz, CA) and *β*-actin (Cell Signaling Technology, Beverly, MA).

### 2.7. Immunofluorescence

Fresh isolated spleens were embedded in OCT buffer and frozen sections were fixed with 4% paraformaldehyde, permeabilized with 0.3% Triton X-100. Then sections were incubated with Nek2 primary antibody or FITC-conjugated PNA overnight. After washing with 1x PBS, Alexa-Fluor 488 or Alexa-Fluor 568 conjugated secondary antibodies (Invitrogen, Carlsbad, CA) were added and incubated for 1 hour at room temperature. Cells were washed with 1x PBS and stained with DAPI. Images were recorded via Zeiss LSM710 confocal fluorescence microscope.

### 2.8. Gene Expression Profiling (GEP)

Messenger RNA was extract from primary cells using RNeasy kit (Qiagen, Valencia, CA) following the manufactures protocol. GEP was performed on the Affymetrix 7 G workstation and data was analyzed using as previously reported [21].

### 2.9. Statistics

A Student's* t*-test was used to evaluate the difference between two different groups with a *P* value less than 0.05 considered statistically significant.

## 3. Results

### 3.1. Generation of Transgenic Mice with B Cell Lineage Specific Overexpression of Nek2

**Figure 1:**
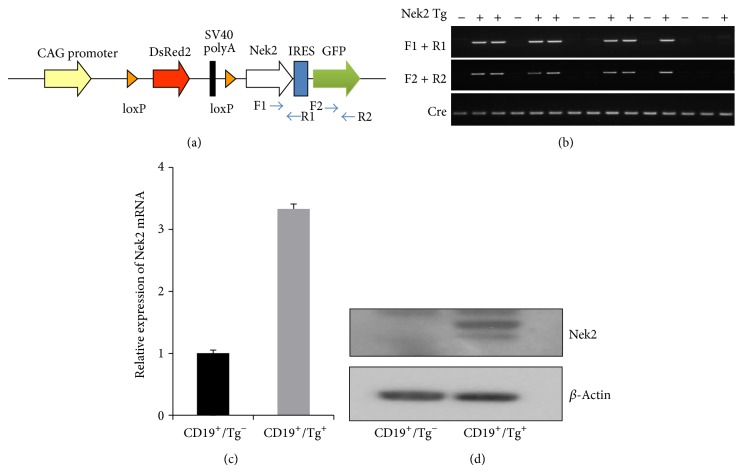
Generation of transgenic mice with B cell lineage specific overexpression of Nek2. (a) Illustration of construct used to generate transgenic mice. (b) Nek2 Tg mice were bred with CD19-Cre mice and genotyped with F1 + R1, F2 + R2, and Cre primers. (c) CD19 positive cells were sorted from transgene positive mice and its littermate controls (*n* = 3). Expression of Nek2 mRNA was detected by RT-qPCR. (d) Expression of Nek2 was detected by western blots in CD19 positive sorted cells.

Nek2 is frequently overexpressed and associated with disease progression in many types of cancer including B cell neoplasm [9]. Here we generated a transgenic mouse model in which expression of Nek2 was conditionally regulated by Cre recombinase expression. To generate the Nek2 expression construct, the Nek2 coding sequence was cloned downstream of floxed DsRed2-SV40 polyA and driven by a CAG promoter (Figure 1(a)). Upon breeding with CD19-Cre, induction of Cre recombinase resulted in the excision of the DsRed2-SV40 polyA fragment and induced the expression of Nek2 and GFP under the control of the CAG promoter (Figure 1(a)). A 9.6 kb Nek2 expression fragment was microinjected into CBA × C57BL6 embryos. Positive founders were genotyped and back-crossed with C57BL6 for more than ten generations to produce mice with a relatively pure C57BL6 background. Transgenic mice were bred with CD19-Cre mice to induce expression of Nek2 in the B cell lineages. C57BL6-Nek2 mice were genotyped using two separate pairs of primers to ensure accuracy and identify transgenic Nek2 mice and their littermates, F1 + R1 against the region containing Nek2-IRES and F2 + R2 against the region containing GFP (Figure 1(c)). To identify Nek2 transgenic mice, a pair of Cre primers were also used (Figure 1(b)). Splenocytes from transgenic mice were labeled with a CD19 antibody and sorted by flow cytometry to identify CD19^+^ cells. We demonstrated that the expression of Nek2 was 3.3-fold higher in cells expressing the transgene compared to cells void of the transgene (Figure 1(c)). Western blots consistently demonstrated an increase in the Nek2 protein levels in mice expressing the Nek2 transgene (Figure 1(d)). These results demonstrate a successful introduction of stable Nek2 expression within the B cell lineage and displayed a significantly higher expression of Nek2 in the Nek2 positive transgenic mice compared to transgenic negative littermates.

### 3.2. Overexpression of Nek2 Increases the Number of Immature B Cells and Decreases the Number of B-2 and B-1a B Cells

**Figure 2:**
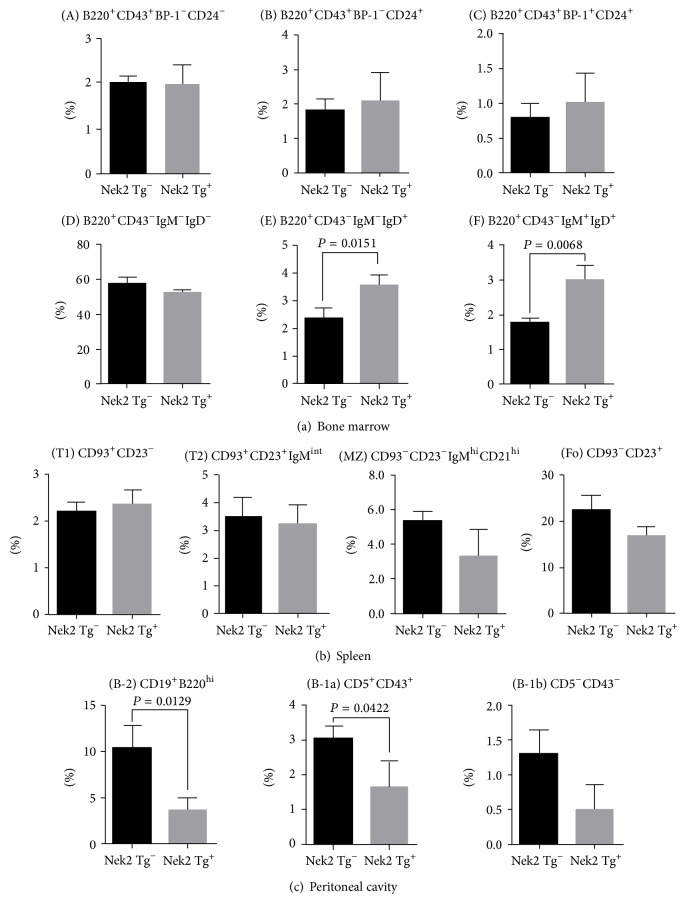
Analysis of B cell development in Nek2 Tg mice. (a) Hardy fractions from bone marrow (fraction (A) to (F)) were gated as follows: (A) B220^+^CD43^+^BP-1^−^CD24^−^; (B) B220^+^CD43^+^BP-1^−^CD24^+^; (C) B220^+^CD43^+^BP-1^+^CD24^+^; (D) B220^+^CD43^−^IgM^−^IgD^−^; (E) B220^+^CD43^−^IgM^−^IgD^+^; and (F) B220^+^CD43^−^IgM^+^IgD^+^. Fractions (A) and (B)–(D) may also be gated as CD19^−^ and CD19^+^ populations among B220^+^IgM^−^ cells, respectively (b) IgM^+^ splenocytes were separated into the following subsets: (T1), CD93^+^CD23^−^; (T2), CD93^+^CD23^+^IgM^int⁡^; marginal zone (MZ), CD93^−^CD23^−^IgM^hi^CD21^hi^; and follicular (Fo), CD93^−^CD23^+^. (c) Peritoneal lymphocytes were separated into the following subsets: (B-2), CD19^+^B220^hi^; (B-1), CD19^+^B220^lo-int^; (B-1a), CD5^+^CD43^+^; and (B-1b), CD5^−^CD43^−^.

Our initial hypothesis focused on Nek2 overexpression exhibiting oncogenic function, in which we anticipated to see an increase in B cell proliferation by ectopic expression of Nek2 in B cell precursors. Elevated expression of Nek2 is a common phenomenon in B cell lymphomas and other neoplastic diseases. However, mice observed for up to 18 months did not display any gross physical or behavioral abnormalities. There were also no changes in the absolute counting of total white blood cells from bone marrow, spleen, and peritoneal cavity (data not shown). We further tested Nek2's oncogenic function by transformation experiments in NIH3T3 cells. Nek2 failed to transform NIH3T3 cells; however, knockdown of Nek2 did attenuate the clonogenic ability of HRAS V12 (data not shown). This data indicated that Nek2 is involved in neoplastic progression but cannot initiate tumorigenesis by itself. This led us to examine whether overexpression of Nek2 plays an important role during B cell development. Therefore, we collected and analyzed B cells from the bone marrow, spleen, and peritoneal cavity to determine any changes of the absolute cell number at each developmental stage from both Nek2 transgenic mice and the control littermates. Not surprising, the absolute cell number of the pre-pro-, pro-, and pre-B cell populations did not exhibit a significant difference between Nek2 transgenic and littermate mice (data not shown). We further analyzed the B cell progenitors in the bone marrow and did not detect any changes in the following transitions; pre-pro-B cell to pro-B cell, Hardy fraction A (B220^+^CD43^+^BP-1^−^CD24^−^), Hardy fraction B (B220^+^CD43^+^BP-1^−^CD24^+^), pro-B to pre-B cell, Hardy fraction C (B220^+^CD43^+^BP-1^+^CD24^+^), or Hardy fraction D (B220^+^CD43^−^IgM^−^IgD^−^) upon the overexpression of Nek2 (Figure 2(a)). Interestingly, we identified a significant increase in the number of immature B cells in the bone marrow (Hardy fraction E, B220^+^CD43^−^IgM^−^IgD^+^ and F, B220^+^CD43^−^IgM^+^IgD^+^) in Nek2 transgenic mice compared to the littermate controls (Figure 2(a)). However, no significant changes were detected in the mature B cell subpopulations isolated from the spleen (Figure 2(b)). Furthermore, analysis of peritoneal lymphocytes revealed that Nek2 transgenic mice had decreased B-2 (CD19^+^B220^hi^) and B-1a (CD19^+^B220^lo-int^) subpopulations while no change in the B-1b (CD5^−^CD43^−^) subpopulation was detected (Figure 2(c)). These data suggest that Nek2 contributes to the early stage of B cell development; however it does not influence late stage B cell development or population expansion.

### 3.3. Nek2 Overexpression Alters TGF*β* and Sox4 Signaling in Immature B Cells

**Figure 3:**
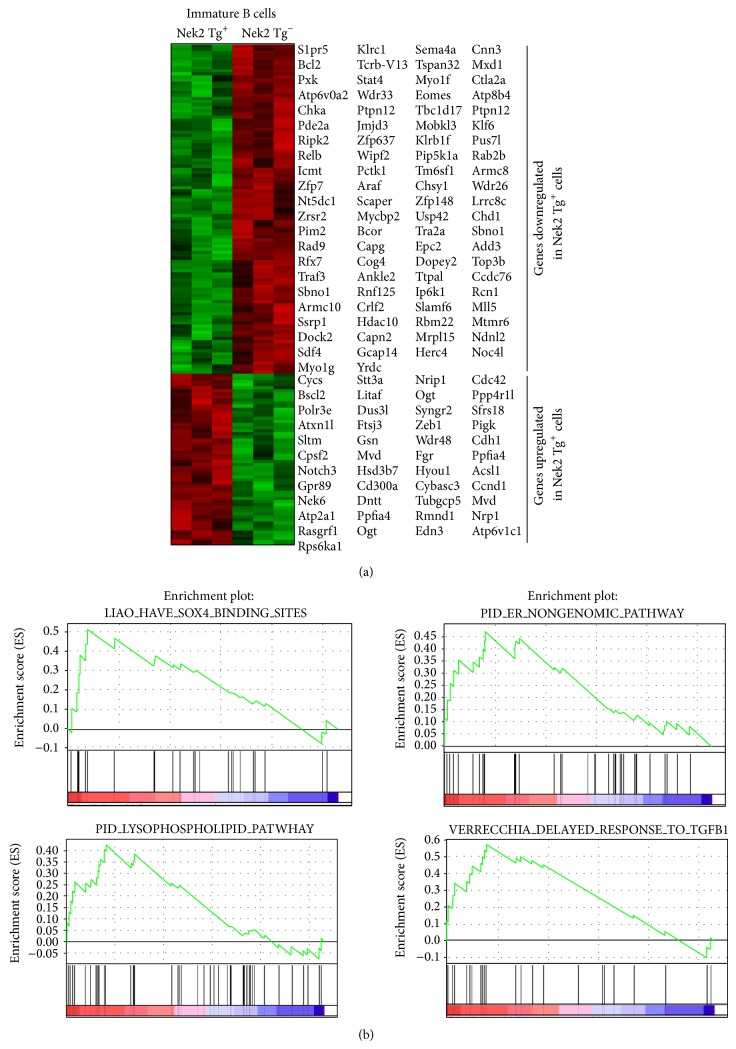
Gene expression profiling of immature, marginal zone and follicular B cells in Nek2CD19-Cre mice and its littermate control. GEP and GSEA analysis of immature B cells ((a) and (b), CD21^−^CD23^−^), sorted from spleen of Nek2 Tg mice and their littermate.

Data presented so far suggests that Nek2 contributes to the development of B cells. This led us to explore signaling pathways related to Nek2 expression to identify pathways that are influenced by Nek2. Gene expression profiling (GEP) arrays were performed on splenic immature B cells from the transgenic Nek2 mice and mice without the transgene present. A total of 846 probe sets, containing 280 with increased and 566 with decreased expression, were detected significantly altered in the Nek2 transgenic mice (*P* < 0.05). One hundred sixty-three significantly altered transcripts with at least 2-fold change are depicted in Figure 3(a) as determined by hierarchical clustering. Furthermore, Gene Set Enrichment Analysis (GSEA) was performed to identify pathways enriched with differentially expressed genes from the Nek2 transgenic mice. Interestingly, we identified a number of pathways involved in B cell development. Of particular interest SOX4, glycosaminoglycan biosynthesis, and delayed response to TGF*β*1 pathways were enriched with genes from mice overexpressing Nek2 (Figure 3(b)). Sox4 has been shown to be an important mediator of early B cell development. Mallampati et al. demonstrate that Sox4 acts as a fine tuner of B cell development by regulating precursor differentiation, thus implicating Sox4 receptors in B cell development [22]. Figure 3(b) identifies the results from GSEA for additional pathways regulated by Nek2 in B cell development. The result from the GEP and GSEA analyses provides additional evidence for the importance of Nek2 expression during B cell development.

### 3.4. Nek2 Overexpression Results in Spontaneous Germinal Center Formation

**Figure 4:**
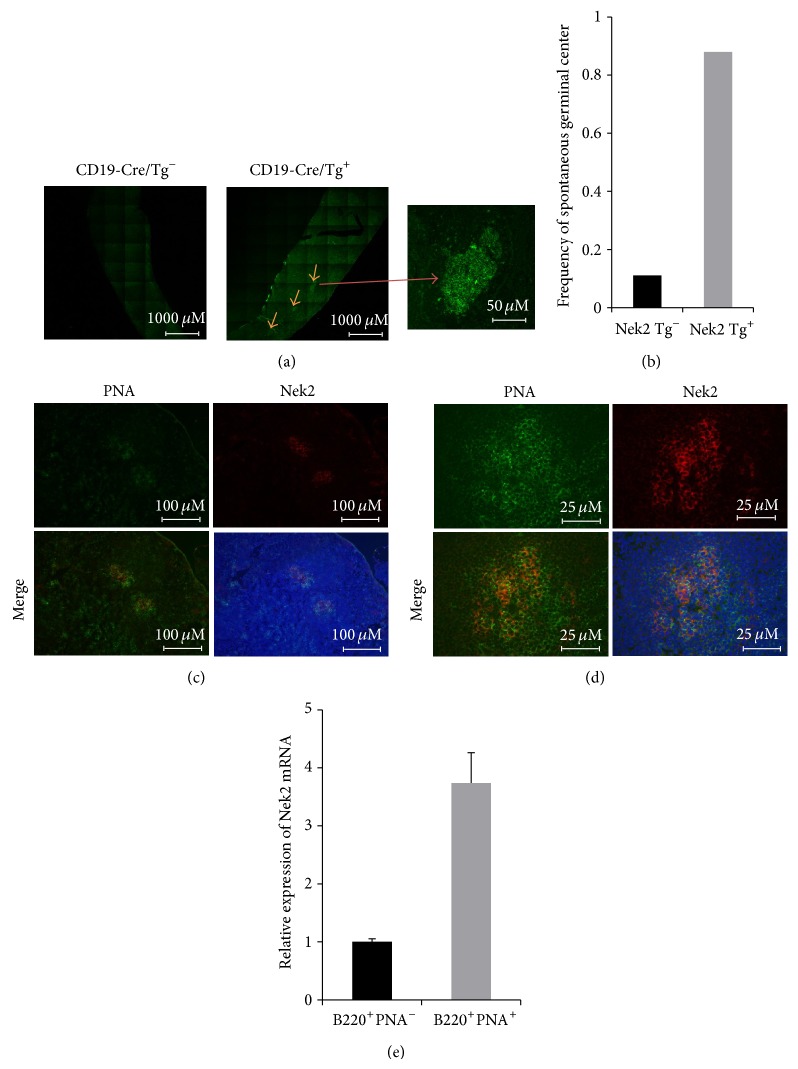
Nek2 is expressed in germinal centers and Nek2 transgene mice develop spontaneous germinal centers. (a) Frozen sections of Nek2 transgenic positive and negative mice were stained with PNA. Sequential images were taken under fluorescence microscope. Spontaneous germinal centers were indicated by arrow and high magnification (right panel). (b) Frequency of spontaneous germinal centers observed in Nek2 transgenic positive and negative mice. ((c)-(d)) C57BL6 mice (*n* = 3) were immunized with SRBC for 10 days. Frozen sections of spleen were stained with Nek2 (Red) and PNA (green). Fluorescence image was taken under 10x (a) and 40x (b). (e) RT-qPCR analysis of Nek2 mRNA expression in B220^+^PNA^−^ and B220^+^PNA^+^ cells sorted from splenocytes of SRBC immunized C57BL6 mice (*n* = 3).

Since no significant change in expansion of the B cell subpopulation in spleen was detected, we next queried the effects of Nek2 overexpression on the formation of germinal centers. Surprisingly, splenic examination detected a significant increase in the number of spontaneous germinal centers formed in the transgenic mice overexpressing Nek2 (8 out of 9 mice, 88.9%) compared to the nontransgenic control mice (1 out of 9 mice, 11.1%) (Figures 4(a) and 4(b)). These results indicated that Nek2 plays an important role in germinal center formation. To determine if a correlation between Nek2 induced germinal center formation and immunologic response exists, C57BL6 mice were immunized with sheep red blood cells (SRBC), a known inducer of germinal center formation, for 10 days followed by quantitative real-time PCR (RT-qPCR) and immunofluorescence microscopy to examine the localization of Nek2 expression. Upon immunization with SRBC, cells were sorted into B220^+^PNA^+^ cells (germinal center associated cells) and B220^+^PNA^−^ cells (nonassociated germinal center cells). The germinal center associated fraction exhibited significantly higher Nek2 expression than the nonassociated germinal center cells when assayed by RT-qPCR and immunofluorescence microscopy (Figures 4(c)–4(e)). These implicate Nek2 is an important mediator of germinal center formation.

### 3.5. Nek2 Enhances the T Cell Dependent Immune Response

**Figure 5:**
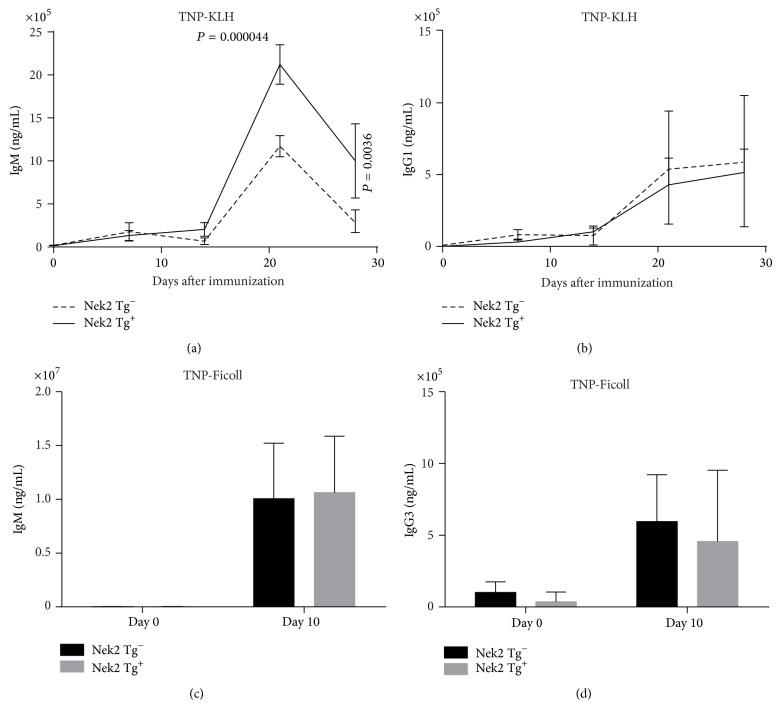
Nek2 enhances T cell dependent immune response* in vivo*. T cell dependent ((a)-(b)) and independent ((c)-(d)) immunization were analyzed by ELISA. Concentration of each specific immunoglobin, as indicated, was calculated using specific standards. For T cell dependent immunization, mice received a boost again at day 14.

Thus far the data implicates Nek2 in early B cell development and germinal center formation, so we pondered about the involvement of Nek2 in the induction of an immune response. Nek2 transgenic and control mice were immunized with a T cell dependent antigen (TNP-KLH) or a T cell independent antigen (TNP-Ficoll) at day 1 followed by a boost at day 14. The Nek2 transgenic mice immunized with the T cell dependent antigen exhibited a significant increase in IgM production compared to the control mice, especially following the day 14 boost (Figure 5(a)). However, there was little difference detected in the IgG1 levels between the transgenic Nek2 mice and the control mice when treated with the T cell dependent antigen (Figure 5(b)). Furthermore, the T cell independent antigen did not induce a significant increase of IgM or IgG3 levels in the Nek2 transgenic mice compared to the control mice (Figures 4(c) and 4(d)). These data implicate Nek2 as a specific regulator of a T cell dependent but not a T cell independent immune response.

## 4. Discussion

We have shown that overexpression of Nek2 promotes tumor cell growth and induces drug resistance in multiple myeloma and serves as a negative clinical prognostic indicator [9]. However, its role in other cellular functions is less clear and requires further work to elucidate. Here we explored the involvement of Nek2 in B cell development. We found, through its overexpression* in vivo*, that Nek2 is an important mediator of early B cell development. Furthermore, we identified Nek2 as an important inducer of germinal center formation and showed that high Nek2 induces an immune response through a T cell dependent mechanism.

Human B cells precursors are derived from hematopoietic stem cells (HSC), located within the bone marrow, through strictly controlled process [23]. Aberrant signal activation may disrupt the developmental program and result in clinically relevant phenotypes like cancer. Transgenic activation of the Pokémon gene in lymphocytes results in T cell arrest at an early stage and can influence the development of T cell lymphoblastic lymphoma/leukemia [24]. Furthermore, a small proportion of BCL6 transgenic mice can also develop T and B cell lymphoma [25]. Although overexpression of Nek2 failed to induce neoplastic growth, Nek2 does contribute to cancer initiation and progression. For instance, Nek2 functions downstream of RAS to induce chromosomal instability, a well-established marker of cancer [16]. Nek2 interacts with and phosphorylates *β*-catenin to regulate centrosome separation, and abnormal expression of Nek2 and *β*-catenin is correlated tumor proliferation in breast cancer [17, 26]. Here we show that activation of Nek2 in a B cell lineage alters B cell development and forms spontaneous germinal centers. Indeed, Nek2 is highly expressed in large diffuse B cell lymphoma which is a postgerminal center cancer [27]. As we previously reported, Nek2 also has a role in the progression of multiple myeloma [9]. These findings suggest that high expression of Nek2 may promote progression of B cell related tumors by itself or cooperating with other oncogenic factors.

Pogue et al. explored the importance of Akt during B cell development. They identify Akt as an important mediator of cell survival during B cell development and a decrease in Akt expression resulted in an increase in the number of apoptotic cells [28]. We previously defined a mechanism for the induction of drug resistance by Nek2 and demonstrated that overexpression of Nek2 activates Akt which results in drug resistance and cellular proliferation in multiple myeloma [9]. It is interesting to speculate about the importance of Akt in the regulation of B cell development induced by Nek2. However, we did not observe any enrichment of Akt regulated genes in immature B cells, indicating that TGF*β* and SOX4 signaling might play more important roles than Akt signaling downstream of Nek2. However, further work using knockout mice is needed to fully elucidate the mechanism of Nek2 downstream signaling in this process.

Germinal center formation is a key process in the maturation of B cells and provides indispensable help to the development of a B cell mediated immune response. Germinal centers are composed of primarily proliferating B cells and to a lesser extent T cells and follicular dendritic cells. Interestingly, we show that Nek2 expression is an important mediator of B cell development and early B cells make up a large portion of cells present within the germinal center potentially explaining why Nek2 expression is high within germinal centers. A potential mechanism of action suggests Nek2 expression promotes early B cell development followed by immature B cell migration into lymphoid follicles and the formation of germinal centers. Since a large proportion of B cells within the germinal center represent immature maturation stages, suggests that Nek2 expression levels would remain high. The results from our study suggest that Nek2 expression results in germinal center formation through its role in mediating early B cell development, including migration to the lymphoid follicles.

B cell activation is a complex process occurring through one of two distinct mechanisms: a T cell dependent and a T cell independent mechanism. As the name applies, T cell dependent requires T cell interaction to activate B cells. Activation occurs when B cells encounter an antigen through the B cell receptor resulting in internalization and degradation of the foreign particle. Peptides from the foreign particle are presented on the cell surface by interactions with the major histocompatibility class II (MHCII) molecule, resulting in the recruitment of T cells and activation of the B cells [29]. Interestingly, Nek2 overexpression does not result in the activation of B cells in a T cell independent fashion (Figures 4(c) and 4(d)) but does influence T cell dependent activation (Figures 4(a) and 4(b)). These results suggest that Nek2 does not alter NF-*κ*B, cell surface receptors, or other mechanisms unique to T cell independent activation or mechanisms shared between the two modes of activation. It would be interesting however to determine if Nek2 alters the concentration of cell surface peptides or if the kinase function is essential for particle internalization or degradation. Further work is required to fully delineate the mechanism of Nek2 in regulating T cell dependent activation.

## 5. Conclusion

Nek2 regulates B cell development* in vivo* by increasing the proportion of immature B cells in the bone marrow, decreasing B-1 B cells in peritoneal cavity and enhances a T cell dependent immune response.
